# Coordinated regulation of core and accessory genes in the multipartite genome of *Sinorhizobium fredii*

**DOI:** 10.1371/journal.pgen.1007428

**Published:** 2018-05-24

**Authors:** Jian Jiao, Meng Ni, Biliang Zhang, Ziding Zhang, J. Peter W. Young, Ting-Fung Chan, Wen Xin Chen, Hon-Ming Lam, Chang Fu Tian

**Affiliations:** 1 State Key Laboratory of Agrobiotechnology, and College of Biological Sciences, China Agricultural University, Beijing, China; 2 Rhizobium Research Center, China Agricultural University, Beijing, China; 3 School of Life Sciences and Center for Soybean Research of the Partner State Key Laboratory of Agrobiotechnology, The Chinese University of Hong Kong, Shatin, Hong Kong SAR; 4 Department of Biology, University of York, York, United Kingdom; Università degli Studi di Firenze, ITALY

## Abstract

Prokaryotes benefit from having accessory genes, but it is unclear how accessory genes can be linked with the core regulatory network when developing adaptations to new niches. Here we determined hierarchical core/accessory subsets in the multipartite pangenome (composed of genes from the chromosome, chromid and plasmids) of the soybean microsymbiont *Sinorhizobium fredii* by comparing twelve *Sinorhizobium* genomes. Transcriptomes of two *S*. *fredii* strains at mid-log and stationary growth phases and in symbiotic conditions were obtained. The average level of gene expression, variation of expression between different conditions, and gene connectivity within the co-expression network were positively correlated with the gene conservation level from strain-specific accessory genes to genus core. Condition-dependent transcriptomes exhibited adaptive transcriptional changes in pangenome subsets shared by the two strains, while strain-dependent transcriptomes were enriched with accessory genes on the chromid. Proportionally more chromid genes than plasmid genes were co-expressed with chromosomal genes, while plasmid genes had a higher within-replicon connectivity in expression than chromid ones. However, key nitrogen fixation genes on the symbiosis plasmid were characterized by high connectivity in both within- and between-replicon analyses. Among those genes with host-specific upregulation patterns, chromosomal *znu* and *mdt* operons, encoding a conserved high-affinity zinc transporter and an accessory multi-drug efflux system, respectively, were experimentally demonstrated to be involved in host-specific symbiotic adaptation. These findings highlight the importance of integrative regulation of hierarchical core/accessory components in the multipartite genome of bacteria during niche adaptation and in shaping the prokaryotic pangenome in the long run.

## Author summary

Prokaryotic pangenomes are characterized by a high rate of turnover in gene content, with core genes shared by all members of a taxonomic group and accessory genes present in only a subset of the members. Accessory functions could serve as an arsenal enabling prokaryotes to develop adaptations to new niches. Therefore, prokaryotic core and accessory components are analogous to the operating system and applications (apps) of smartphones. However, it is puzzling how these accessory functions are linked with the core regulatory network in prokaryotes during niche adaptations. Here we address this question by investigating the adaptive regulation of hierarchical core/accessory subsets in the multipartite pangenome (chromosome, chromid and plasmid) of *Sinorhizobium fredii*, which is a facultative microsymbiont of soybeans. The level and variation of gene expression, and gene connectivity revealed in transcriptomes under free-living and symbiotic conditions are positively correlated with the gene conservation level, i.e. from strain-specific accessory genes to genus core. Replicon-dependent organization and adaptive regulation of hierarchical core/accessory subsets suggest distinct roles of different replicons not only in environmental adaptation but also intra- and inter-species differentiation. Among core and accessory genes with host-specific upregulation patterns, we experimentally identified novel symbiotic players involved in host-specific adaptation.

## Introduction

Prokaryotes play important roles in recycling nutrients and forming pathogenic or mutualistic associations with eukaryotes. It has been established that many ecologically important processes are differentially mediated by prokaryotes at the strain level [1]. This is partially explained by the fact that even closely related strains of bacteria and archaea can have great differences in their genomes due to a high rate of turnover in gene content, so that there are core genes shared by all members of a taxonomic group and accessory genes present in only a subset of the members [2,3]. However, it is still puzzling why and how prokaryotes maintain such a high degree of genome content variability [4]. It is widely accepted that certain accessory genes can benefit their host by conferring the ability to occupy new niches, despite the existence of putative junk genes in the pangenome [4,5]. However, it is largely unexplored to what extent these accessory functions are linked with the core regulatory network during the development of adaptations to new ecological niches.

Soil bacteria able to form nitrogen-fixing nodules on legumes, collectively called rhizobia, have global impacts on sustainable agriculture and the nitrogen cycle. These facultative microsymbionts need a cluster of key symbiosis genes called *nod*/*nif*/*fix*, which are located on a horizontally transferable plasmid or a genomic island, to establish a mutualistic interaction with legume plants [6–10]. The ability to form nitrogen-fixing nodules on legumes has been reported for hundreds of species in alpha- and beta-proteobacteria [11]. Among the 122 complete genome sequences from twelve genera of rhizobia available in the GenBank database (on March, 30^th^ 2018), 107 genomes from eleven genera have two or more DNA molecules, a genome architecture described as a multipartite genome. This multipartite organization is found in approximately 11% of 1,708 bacterial genomes analyzed in a recent study [12]. Each DNA molecule with a separate origin of replication in bacterial genomes is referred to as a replicon. The largest replicon, with most of the core genes, is known as a chromosome, while megaplasmids (above 350 kb in size) and plasmids refer to replicons lacking core genes and are characterized with significantly biased signatures such as GC content and dinucleotide composition compared to the chromosome [12,13]. The term “chromid” was recently introduced to refer to a replicon with plasmid-type maintenance and replication systems, but carrying some core genes and having sequence signatures more similar to chromosomes than plasmids and megaplasmids [12,13]. Accumulating evidence has suggested distinct roles of different replicons in rhizobial adaptations to either saprophytic or symbiotic conditions [14–17], though the coordinated regulation of core and accessory functions in these multipartite genomes is largely unexplored.

A multipartite genome, composed of at least a chromosome, a chromid, and a megaplasmid (the symbiosis plasmid), is present in most sequenced genomes within the *Sinorhizobium* genus, which includes microsymbionts associated with the important legume crops alfalfa and soybean [18–20]. The chromid genes in *Sinorhizobium* associated with the same legume host show a higher differentiation level compared to the other two replicons [21,22]. In contrast to the symbiosis plasmid, which shows evidence of horizontal gene transfer, the chromid core genes have a phylogeny generally congruent with that of chromosomal core genes [21]. An engineered chromosome containing essential core genes transferred from the chromid is sufficient for growth of a model microorganism *Sinorhizobium meliloti* in a sterile bulk soil environment [16]. Metabolic modeling suggests that the chromosome of *S*. *meliloti* also contributes to fitness in rhizosphere, and the chromid shows a greater fitness contribution in the rhizosphere than in bulk soil [15,22]. By contrast, transcriptomics studies of free-living and symbiotic *Sinorhizobium* strains have demonstrated a specific up-regulation of many genes on the symbiosis plasmid within legume nodules, where core functions are generally down-regulated consistent with the growth arrest status of nitrogen-fixing rhizobia [23–25]. However, scattered genetic evidence suggests that genes located on the chromosome and the chromid can also contribute to the integration and optimization of symbiotic functions in diverse rhizobia including *Sinorhizobium* [25–30]. It has been proposed that the rhizobium-legume symbiosis requires optimization through a long-term evolutionary process involving integration of lineage-specific accessory genes (those genes only present in a limited subset of related strains, species or genera) with the regulatory network of core genomes [26,31], but there is little direct evidence as yet [30,32]. There is a need for omics-based comparative analyses of the variation in the contents, regulation and integration of core and accessory genes under different conditions.

In this study, we investigate how core and accessory genes are organized and integrated in the multipartite genome of the soybean microsymbiont, *Sinorhizobium fredii*. To this end, complete genome sequences were obtained for *S*. *fredii* CCBAU45436 and CCBAU25509, which have an overlapping host range. The genes of these two genomes were divided into four hierarchical core/accessory subsets based on comparative genomics analyses with ten published genomes of *Sinorhizobium* spp. Then the global transcriptomic profiles of the two test strains were determined at exponential and stationary phases in free-living cultures, and at the symbiotic stage within the nodules of cultivated and wild soybeans. By analyzing this transcriptomic and genomic information, we obtained a global integration pattern of core and accessory genes under different conditions, and identified novel genes involved in symbiotic adaptations. These findings will be discussed in the more general context of the organization and evolution of the prokaryotic pangenome in relation to ecological adaptations.

## Results

### Host specificity and multipartite genomes of the two soybean microsymbionts

**Fig 1:**
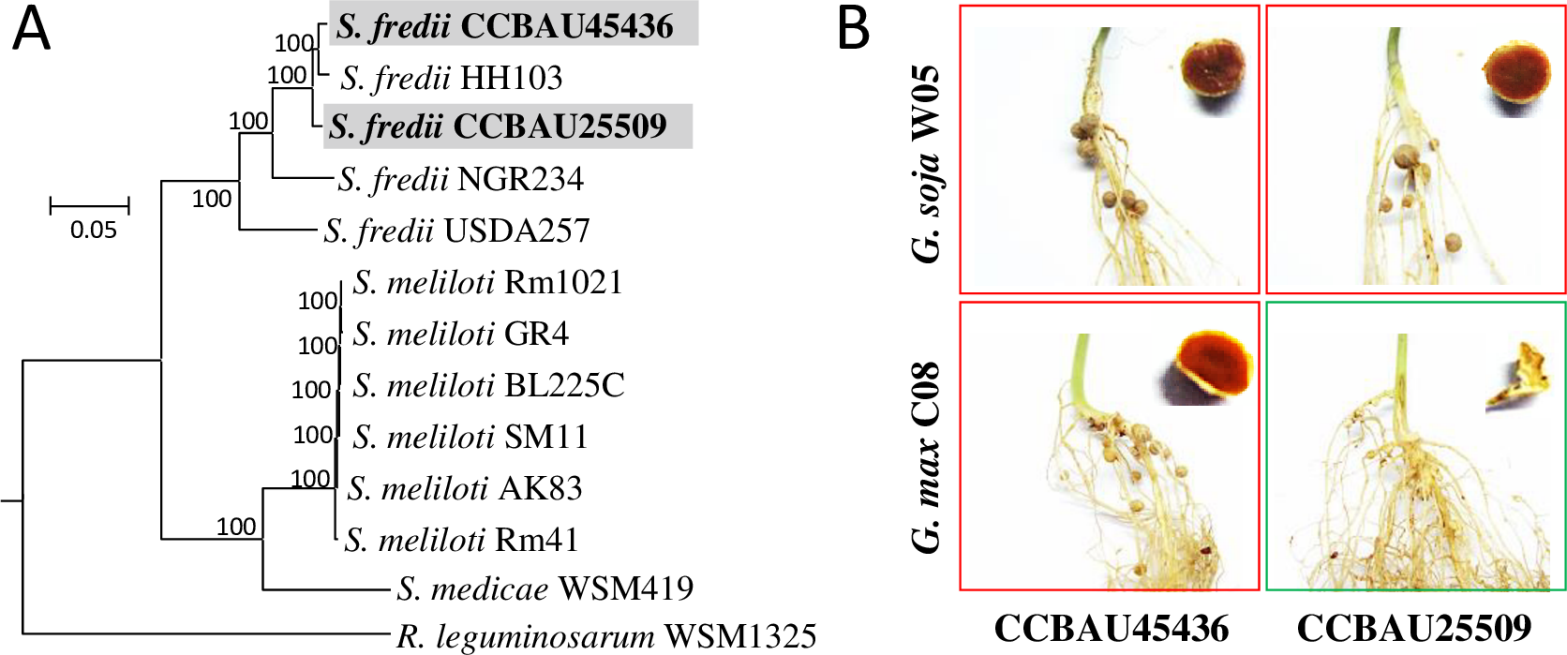
Phylogeny and symbiotic performances of *S*. *fredii* CCBAU45436 and CCBAU25509. (**A**) A maximum likelihood phylogenetic tree based on the concatenated 1278 core genes shared by *Sinorhizobium* strains and an outgroup strain *Rhizobium leguminosarum* WSM1325. Scale bar represents 5% substitutions per site. (**B**) Effective nodules and/or pseudonodules induced by test strains on *Glycine soja* W05 and *Glycine max* C08. The red color of nodule sections indicates effective nitrogen-fixing nodules.

*S*. *fredii* CCBAU45436 and CCBAU25509 (Fig 1A), which are effective microsymbionts of local soybean cultivars grown in northern China [33], induced normal nitrogen-fixing nodules and non-fixing nodule-like structures, respectively, on the roots of soybean accession C08 (Fig 1B), which is a close relative of the sequenced soybean cultivar Williams 82 [34,35]. They both established nitrogen-fixing nodules on the wild soybean accession W05 (Fig 1B), which has recently been sequenced [36].

Complete genome sequences for CCBAU45436 and CCBAU25509 were first obtained by assembling Illumina data generated previously [26]. In this study, full assembly of these genomes were achieved by new PacBio and Ion Torrent sequencing data (S1 Table), and Sanger sequencing of PCR products was used to fill assembly gaps when necessary. The general features of CCBAU45436 and CCBAU25509 genomes are summarized in S2 Table. CCBAU25509 has a typical tripartite genome, consisting of a chromosome (cSF25509; 4.20 Mb), a chromid (pSF25509b; 2.21 Mb) and a symbiosis plasmid (pSF25509a; 0.40 Mb). In the CCBAU45436 genome, two additional smaller plasmids, pSF45436d (0.20 Mb) and pSF45436e (0.17 Mb) were also found besides the chromosome (cSF45436; 4.16 Mb), the chromid (pSF45436b; 1.96 Mb) and the symbiosis plasmid (pSF45436a; 0.42 Mb).

**Fig 2:**
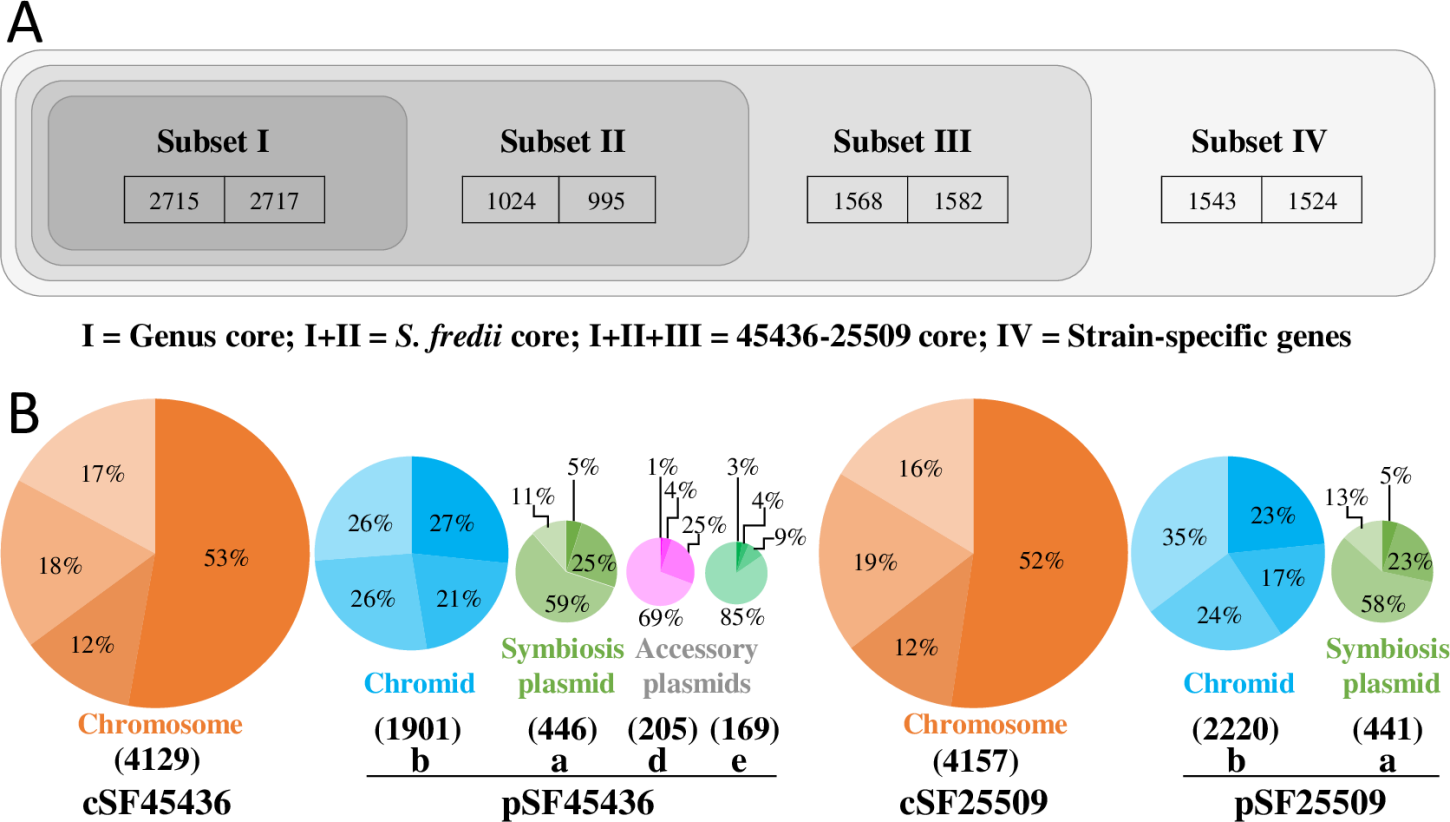
Biased distributions of core and accessory genes regarding replicons. **(A)** A schematic diagram illustrating the hierarchical division of core/accessory subsets for the genomes of *S*. *fredii* CCBAU45436 and CCBAU25509. Subset I, genus core genes present in all strains; subset II, genes present in all *S*. *fredii* strains excluding subset I; subset III, genes shared by CCBUA45436 and CCBAU25509 but not present in all *S*. *fredii* strains, i.e. excluding subsets I and II; subset IV, the remaining accessory genes in either CCBAU45436 or CCBAU25509. The actual total gene numbers of each subset within the genome of CCBAU45436 (left) and CCBAU25509 (right) are shown in frames. The slight differences between two strains in the numbers of core genes in subsets I-III are due to the counting of multi-copy genes belonging to the same homologous gene cluster, which can include one or more genes in each strain. **(B)** Percentage values are the ratios of genes included in hierarchical core/accessory subsets (shades of colors from deep to light: I, II, III, IV), harbored by each replicon within the genome of CCBAU45436 or CCBAU25509. The actual total gene numbers of each replicon are shown in parentheses. Pearson's chi-square test of independence indicates the distribution of different core/accessory subsets on replicons is not random (CCBAU45436, X-squared = 1455.3, df = 12, *P* < 2.2E-16; CCBAU25509, X-squared = 1010.4, df = 6, *P* < 2.2E-16).

By including ten published genomes of *Sinorhizobium* (Fig 1A and S1 Fig), the gene homologs shared by CCBAU45436 and CCBAU25509 were each divided into three hierarchical core subsets (Fig 2A): subset I, gene homologs present in all *Sinorhizobium* strains; subset II, those present in all *S*. *fredii* strains excluding subset I; subset III, those shared by CCBUA45436 and CCBAU25509 but not present in all *S*. *fredii* strains, i.e. excluding subsets I and II. The remaining accessory genes of CCBAU45436 or CCBAU25509 were defined as subset IV. As expected, genes within each of these hierarchical core/accessory subsets were unevenly distributed on different replicons in the two strains (Fig 2B and S3 Table; Pearson’s chi-square test, *P* < 0.001). Around 80% of the subset I genes were concentrated on chromosomes. Genes within subsets II and IV were overrepresented on chromids. The symbiosis plasmids were characterized by their enrichment with the subset III genes (58%-59% genes on the symbiosis plasmid) and to a lesser extent with the subset II genes (23%-25%). Two replicons (pSF45436d and pSF45436e) specific to CCBAU45436 were extremely enriched with the subset IV genes (69.3% and 84.6%).

### Replicon-dependent transcriptional profiles of genes within different hierarchical core/accessory subsets under free-living and symbiotic conditions

**Fig 3:**
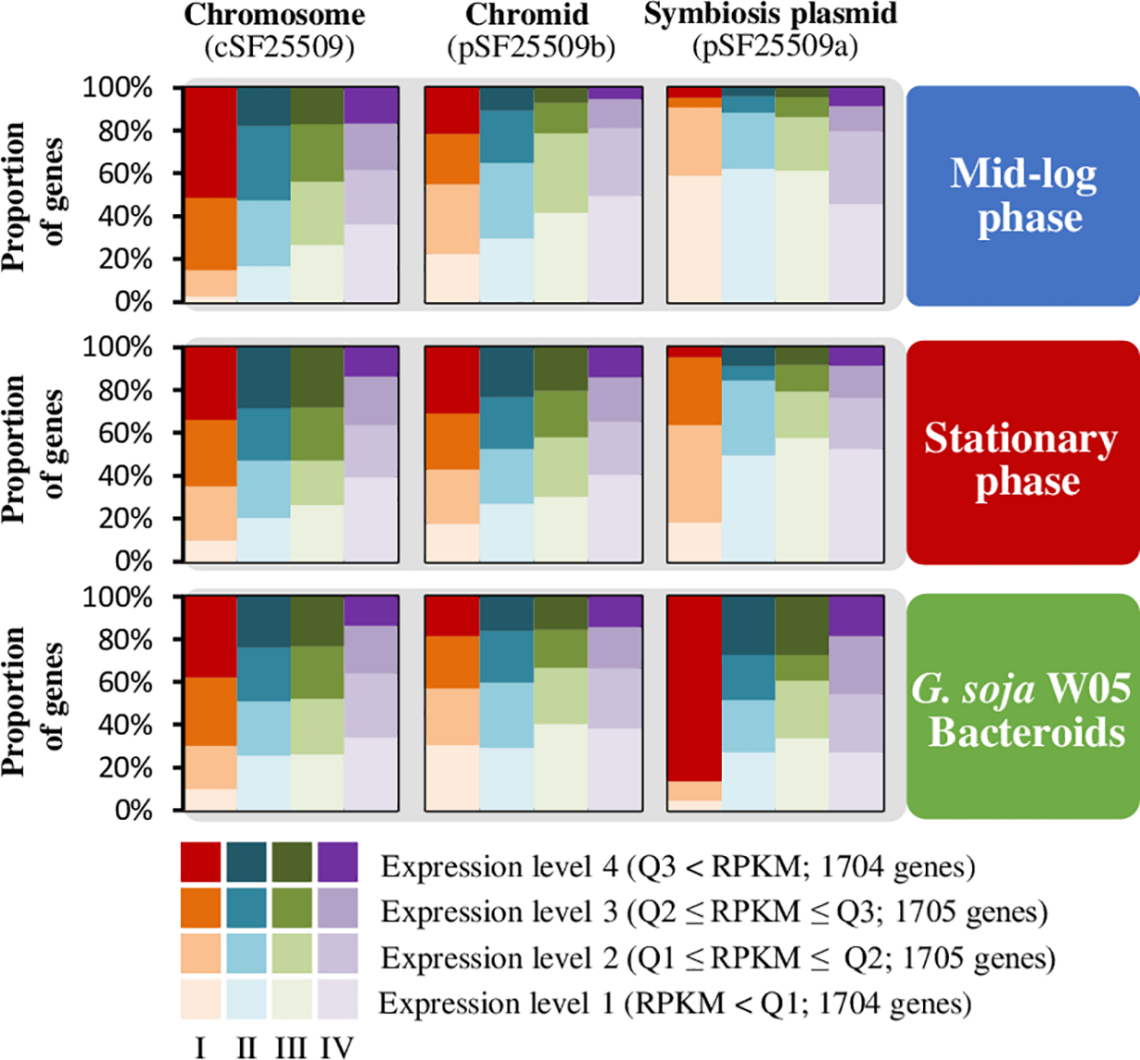
Replicon-dependent transcriptional profiles of genes within different hierarchical core/accessory subsets under free-living and symbiotic conditions. Bar intensities represent the proportion of genes transcribed at four expression levels (arbitrary cut-offs at the first, second and third quartiles of expression profiles based on RPKM values of all genes) for each of the four hierarchical core/accessory subsets (I-IV) under free-living (mid-log phase and stationary phase) and symbiotic conditions (bacteroids isolated from *G*. *soja* W05 nodules). Q1, Q2 and Q3 represent the first, second and third quartiles, respectively. Results are shown separately for each of the three replicons (chromosome, chromid, plasmid) in the genome of CCBAU25509. Log-linear analyses indicate significant differences among the replicons in the distribution of genes of different transcriptional levels for each of the hierarchical core/accessory subsets under each condition (all *P* < 0.001). Similar replicon-dependent expression pattern was also found in CCBAU45436 (see **S2 Fig**).

To investigate how core and accessory genes with biased replicon distributions were integrated during adaptations, we used RNA-seq to obtain transcriptomes of the two test strains under three conditions: (1) free-living culture in the mid-log phase (non-stress), (2) free-living culture in the nutrient-starved stationary phase (abiotic stress), and (3) symbiotic bacteroids within the nodules of cultivated and/or wild soybeans (biotic stress) (S4 Table). For convenience, genes were classified into four expression levels (*Level_1-Level_4*) using arbitrary cut-offs at the first, second and third quartiles of the expression profiles based on the RPKM (reads per kilobase per million mapped reads) value of each gene under test condition. The distribution of these genes across different transcriptional levels under test conditions was analyzed for each replicon (Fig 3 and S2 Fig).

On the chromosomes and chromids, the proportion of genes expressed at levels higher than the first quartile (above *Level_1*) decreased along with reduced gene conservation levels (from subset I to subset IV) under all test conditions (Fig 3 and S2 Fig). This phenomenon can also be found in the transcriptional profiles of symbiosis plasmid genes under symbiotic conditions but not in free-living cultures, particularly for highly expressed genes (*Level_4*). There was generally an increased number of highly expressed genes (*Level_4*) in subsets I-IV of the symbiosis plasmid in legume nodules compared to free-living cultures. By contrast, the proportion of high-expressed (*Level_4*) subset I genes on the chromosome was notably reduced under symbiotic conditions and in the stationary phase compared to that of mid-log phase. The chromid genes did not exhibit drastic changes in the proportions of different transcriptional levels under test conditions, except a notable increase of highly expressed genes (*Level_4*) at the stationary phase compared to the mid-log phase. Although transcriptional levels showed a strong dependence on both the replicon location and the conservation levels, log-linear analysis indicated that replicon and core/accessory status were independently related to gene expression levels (all *P* < 0.001).

### Transcriptional profiles of core genes reflect environmental adaptations

**Fig 4:**
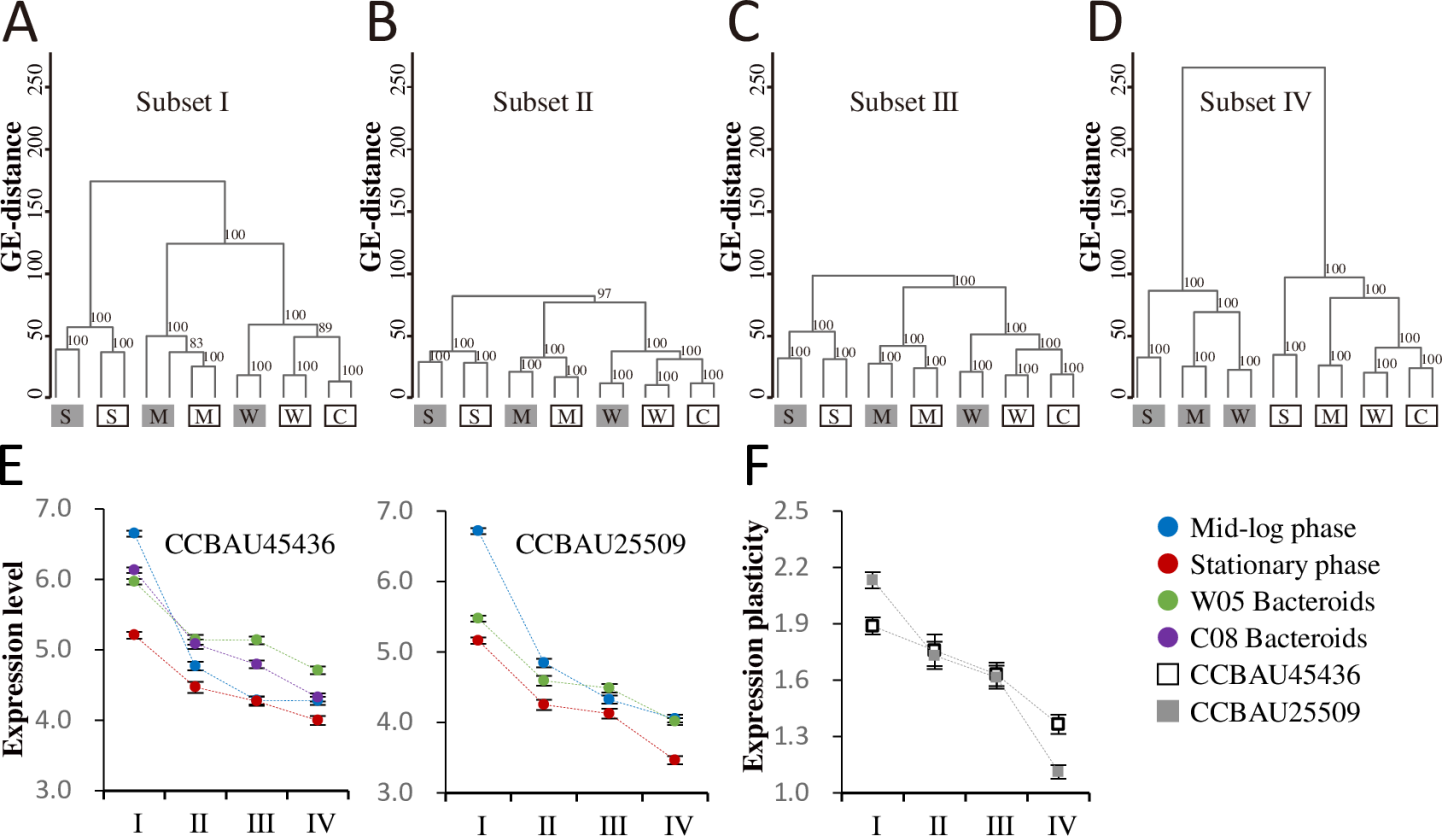
Condition-dependent transcriptional profiles of *S*. *fredii* CCBAU45436 and CCBAU25509 under free-living and symbiotic conditions. (**A-D**) Clustering analyses of Log_2_-transformed RPKM values for genes within different hierarchical core/accessory subsets (I-IV) using the average linkage method based on Euclidean distance. CCBAU45436 and CCBAU25509 are represented by white and grey boxes respectively. S, stationary phase; M, mid-log phase; W/C, microsymbionts in nodules of *G*. *soja* W05 or *G*. *max* C08. Bootstrap values above 70% are indicated. (**E**) Gene expression level within different hierarchical core/accessory subsets under test conditions, dots and error bars refer to the means and standard errors of Log_2_-transformed RPKM values. (**F**) Gene expression plasticity within different hierarchical core/accessory subsets under test conditions, dots and error bars refer to the means and standard errors of variances of Log_2_-transformed RPKM values. Multi-copy genes were not included in these analyses.

To further investigate how genes within different hierarchical core/accessory subsets would respond to different growth conditions, dendrograms based on gene expression distance (GE distance, defined in Materials and Methods) were constructed. When we examined the expression profiles of shared genes within each of subset I, subset II and subset III, the profiles of the two strains were closely matched with respect to growth phases and symbiotic conditions (Fig 4A–4C), while the expression profiles of the strain-specific genes (subset IV) were, inevitably, clustered by strain (Fig 4D). The overall picture is that, for all gene subsets, expression in nodules is more similar to expression in exponential phase than in stationary phase and, for all subsets that they share, the difference between the two strains is less than the effect of growth conditions.

Although similar condition-dependent clustering patterns were observed for subsets I-III (Fig 4A–4C), the average gene expression level under each condition decreased with reduced gene conservation level (from subset I to subset IV) (Fig 4E). Moreover, the higher expression plasticity (gene expression variance among conditions) was observed for the more conserved subsets (Fig 4F), and subset IV showed the least variance in expression plasticity. As expected, further analyses of the differentially expressed genes (DEGs, Log_2_R > 1.732, FDR < 0.001) based on pairwise comparisons showed that DEGs were significantly enriched in subset I and/or subset II, while depleted in subset III and/or subset IV (S5 Table, all *P* < 0.05). It is noteworthy that up-regulated and down-regulated genes had distinct enrichment patterns across the core/accessory subsets (S3 Fig and S5 Table). Genes down-regulated at the stationary phase or in the symbiotic nodules compared to the mid-log phase were enriched in subset I (the genus core genes), while the up-regulated ones were enriched in subsets II and III (the genus accessory genes shared by the two test strains) (Pearson’s chi-square test, all *P* < 0.05). These results provided another line of strong evidence for differential roles of core genes with different conservation levels during environmental adaptation. To further dissect this phenomenon, we then examined the condition-dependent co-expressed genes.

### Distinct groups and functional categories of genes are involved in environmental adaptations

**Fig 5:**
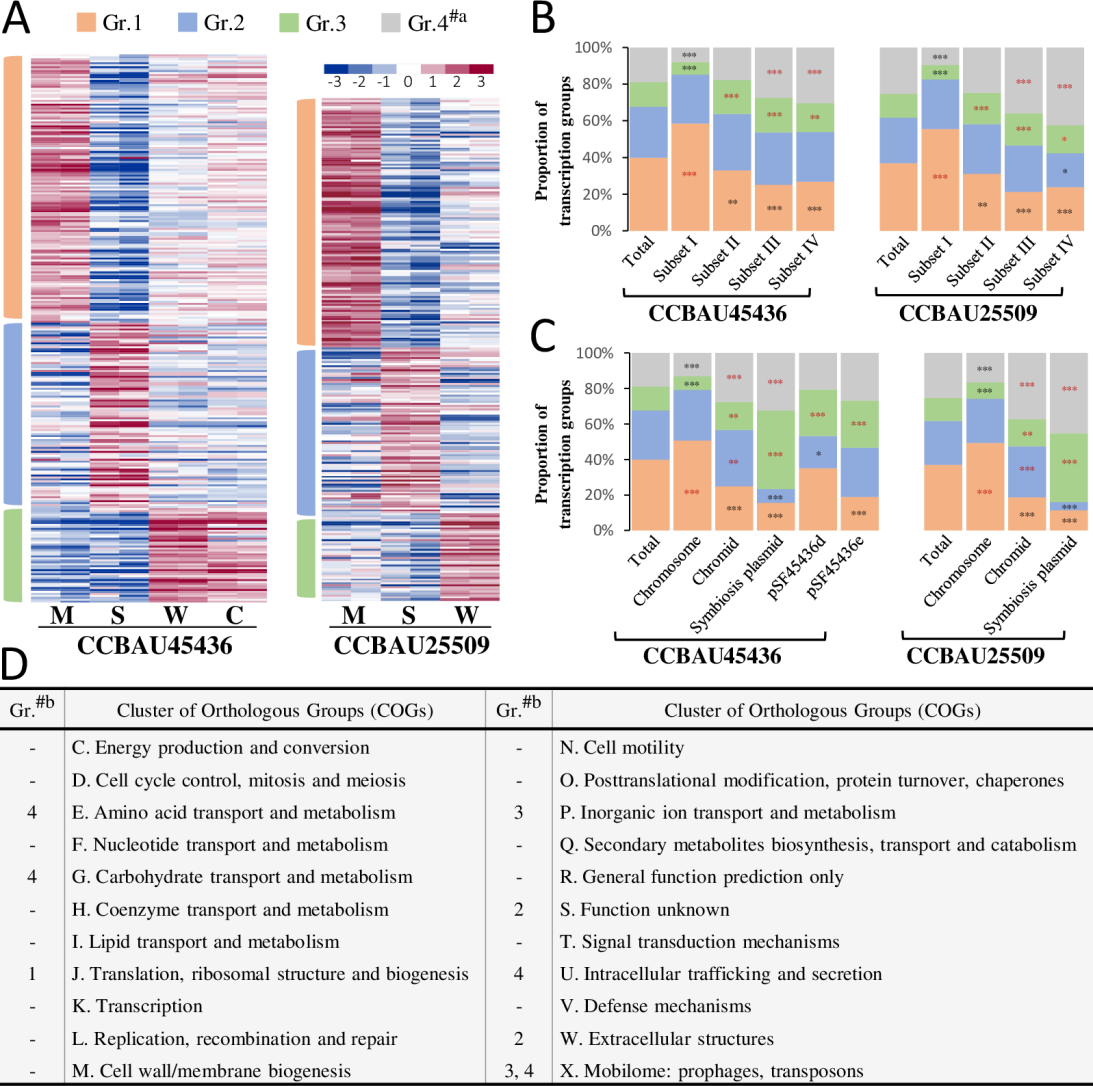
Biased distributions of genes in condition-dependent transcription groups with respect to replicons, core/accessory subsets and COG categories. (**A**) K-means clustering of transcriptional profiles of 5,561 (CCBAU45436) and 5,101 (CCBAU25509) genes that were induced under at least one condition (RPKM > 15, Log_2_R > 1). S, stationary phase culture; M, mid-log phase culture; W, microsymbionts in nodules of wild soybean W05; C, microsymbionts in nodules of cultivated soybean C08. The color of scale bar indicates the relative level of gene expression. #a, Group 4 includes the genes expressed constitutively under all conditions. (**B-C**) Enrichment analyses of condition-dependent transcription groups for each of the replicons and core/accessory subsets (I-IV). Significant enrichment/depletion is indicated by red/black stars (*, *P* < 0.05; **, *P* < 0.01; ***, *P* < 0.001; Pearson’s chi-square test). (**D**) Distribution of COG categories among condition-dependent transcription groups. #b, Groups significantly enriched in the corresponding COG categories for both CCBAU45436 and CCBAU25509 (all *P* < 0.05, Pearson’s chi-square test).

Genes could be divided into four groups based on k-means clustering of their transcriptional profiles (*Gr*.*1-4*; Fig 5A). *Gr*.*4* consisted of genes constitutively expressed or non-expressed under all conditions, while *Gr*.*1*, *Gr*.*2* and *Gr*.*3* consisted of those up-regulated at mid-log phase, stationary phase and symbiotic stage in nodules respectively (Fig 5A). Genes within different condition-dependent groups were unevenly distributed in the hierarchical core/accessory subsets (Fig 5B). *Gr*.*4* was overrepresented within subsets III and IV (Pearson’s chi-square test, all *P* < 0.001). *Gr*.*1* genes were enriched in subset I, *Gr*.*3* genes in subsets II-IV, while *Gr*.*2* genes in none of them (Fig 5B). Among different replicons, the chromosomes and symbiosis plasmids were enriched with *Gr*.*1* genes and *Gr*.*3* genes, respectively, while both *Gr*.*2* and *Gr*.*3* genes were overrepresented on the chromids (Fig 5C), indicating a replicon-dependent gene regulation under test conditions.

Functional annotations of genes within *Gr*.*1-4* were further analyzed regarding COG categories. *Gr*. *1*, *Gr*.*2* and *Gr*.*3* were respectively enriched in the COG category *J* (translation, ribosomal structure and biogenesis), *S/W* (*S*: function unknown; *W*: extracellular structures) and *P/X* (*P*: inorganic ion transport and metabolism; *X*: mobilome: prophages, transposons) (Fig 5D).

### Strain-dependent DEGs are enriched on chromids and overrepresented by the intraspecies accessory genes shared by CCBAU45436 and CCBAU25509

**Fig 6:**
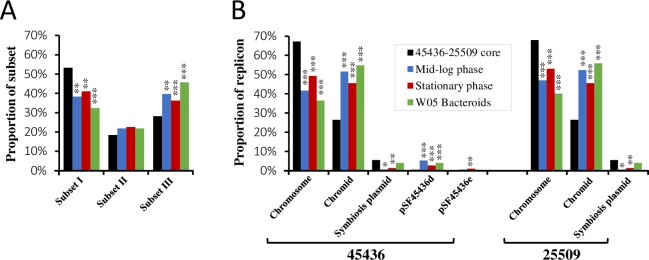
Replicon-dependent transcriptional variations between CCBAU45436 and CCBAU25509 under free-living and symbiotic conditions. (**A**) Enrichment analyses of differentially expressed genes (Log_2_R > 1.732, FDR < 0.001) between CCBAU45436 and CCBAU25509 under free-living and symbiotic conditions for the hierarchical core/accessory subsets. (**B**) Enrichment analyses of differentially expressed genes between CCBAU45436 and CCBAU25509 under free-living and symbiotic conditions for each replicon. The black bars represent the background ratios of 45436–25509 shared genes in each of the hierarchical core/accessory subsets or replicons. Significant enrichment and/or depletion are indicated by stars (*, *P* < 0.05; **, *P* < 0.01; ***, *P* < 0.001; Pearson’s chi-square test). All analyses of this figure are based on datasets without multi-copy genes and strain-specific genes.

Among the 4,931 single-copy orthologous genes shared by CCBAU45436 and CCBAU25509, the DEGs between these two strains (151 at the mid-log phase, 292 at the stationary phase, and 197 within the nodules of *G*. *soja* W05; Log_2_R > 1.732, FDR < 0.001) were significantly enriched in the hierarchical core/accessory subset III (Fig 6A and S6 Table). This provides further evidence that the differential regulation of intraspecies accessory genes may contribute to bacterial diversification. Consistent with results described above that genes within different hierarchical core/accessory subsets exhibited a biased replicon distribution pattern (Fig 2), the strain-dependent DEGs were significantly enriched on the chromids, and the non-symbiosis plasmid pSF45436d (Fig 6B & S6 Table).

### Positive relationship between gene connectivity and gene conservation levels in *S*. *fredii* genomes

**Fig 7:**
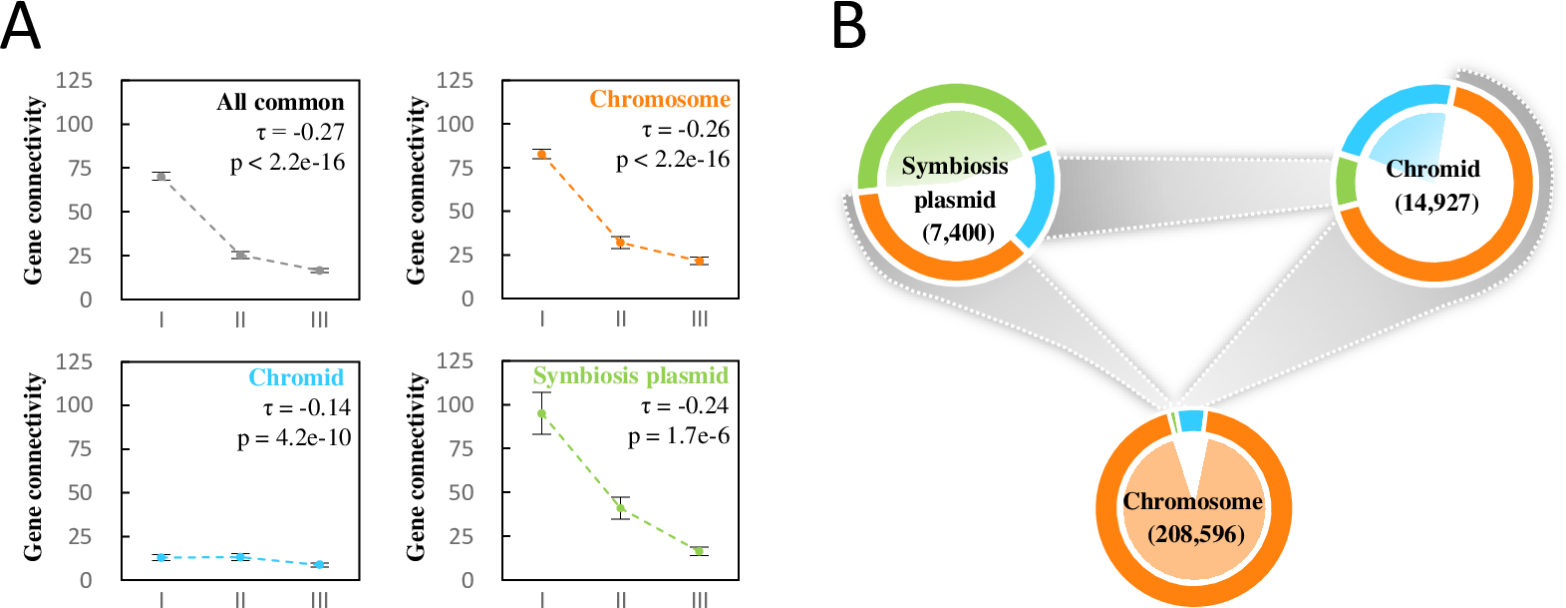
Connectivity analyses of gene co-expression networks in the multipartite genome of *S*. *fredii* CCBAU25509. **(A)** Gene connectivity degrees of the *Sinorhizobium* pangenome subsets for each replicon. Error bars represent standard error of mean. **(B)** Within- and between-replicon gene connectivity. The total number of gene connectivity identified for each replicon is shown in brackets. The relative abundances of within- and between-replicon gene connectivity are indicated by different sections of the perimeter colored according to the connected replicons (orange, the gene connectivity to the chromosome cSF25509; blue, the chromid pSF25509b; light green, the symbiosis plasmid pSF25509a). Between-replicon gene connectivity is depicted in grey. The within- and between-replicon connective patterns of CCBAU45436 (see **S4 Fig**) are similar to those of CCBAU25509 shown here. Multi-copy genes except one out of two *nifHDK* copies were not included in these analyses.

The biased distribution of condition-dependent co-expressed genes and strain-dependent DEGs with respect to core/accessory genomes and replicons raised the question of whether accessory genes have been integrated in a replicon-dependent way among *S*. *fredii* strains. Therefore, we investigated the gene connectivity (co-expression of gene pairs) within or between replicons in gene co-expression networks constructed from the transcriptional profiles of *S*. *fredii* CCBAU45436 and CCBAU25509 (described in Materials and Methods). When the genes from all replicons were pooled together, a significant decrease in gene connectivity was revealed in parallel with the decreasing conservation level of the genes (from subset I to subset III) (Fig 7A and S4 Fig). This correlation was observed on chromosomes and symbiosis plasmids, but not on chromids and other plasmids (pSF45436d/e) (Fig 7A and S4 Fig).

A larger fraction (68%) of chromid genes were linked to the chromosome than were the symbiosis plasmid genes (36%) (Fig 7B and S4 Fig), indicating that chromids are more closely associated with chromosomes than symbiosis plasmids in terms of transcriptional regulation. On the other hand, the symbiosis plasmid possessed a larger fraction (46%) of within-replicon gene connectivity than the chromid (23%) (Fig 7B and S4 Fig), and most of the within-replicon gene connectivity on the symbiosis plasmid was linked to genes required to support symbiotic nitrogen fixation, such as *nif* and *fix* genes (S5 Fig). Nevertheless, more than half (54%) of the gene connectivity associated with the symbiosis plasmid was between-replicon (Fig 7B and S4 Fig). Both the typical symbiosis genes with high within-replicon gene connectivity and certain genes with low within-replicon gene connectivity can show a high level of between-replicon gene connectivity (S5 Fig). These genes with between-replicon connectivity could be interesting candidates for further functional analyses of the optimization of symbiosis.

### Identification of chromosomal loci involved in host adaptations

CCBAU45436 can form effective nodules on both the wild soybean, *G*. *soja* W05, and the cultivated soybean, *G*. *max* C08. This allowed us to investigate the potentially adaptive transcriptional profiles of rhizobia in the nodules of a cultivated soybean compared to those in wild soybean nodules. There were 42 and 77 genes down-regulated and up-regulated, respectively, in CCBAU45436 bacteroids within C08 nodules compared to those in W05 nodules (Log_2_R > 1, FDR < 0.001; S1 Dataset). These DEGs were slightly enriched in the subset II (harboring 24.4% of DEGs and 14.9% of the total number of genes; Pearson’s chi-square test, *P* < 0.05) but were not enriched in any one of the replicons.

To uncover potential candidate genes essential for host adaptation, we constructed mutants for ten representative genes (S6 Fig and S7 Table) that were up-regulated in C08 nodules compared to W05 nodules. These representative genes were among those with the highest log_2_R values and covered the four conservation levels (subsets I-IV; S1 Dataset). Eight of the mutants exhibited indistinguishable symbiotic phenotypes on both W05 and C08 compared to the wild type (S8 Table), but Δ*znuA* and *mdtA*::pVO had significant effects (Table 1). The *Sinorhizobium* core genes *znuA/B/C* (in subset I) encode the conserved zinc transporter components, and the in-frame deletion mutant of *znuA* (Δ*znuA*) formed a reduced number of nodules (34.9% - 48.4%, respectively, compared to wild type, *P* < 0.01) on both W05 and C08, but with higher fresh weight per nodule (167% - 247% of wild type, respectively, *P* < 0.05) (Table 1 and S7 Fig). C08 plants nodulated by Δ*znuA* had lower leaf chlorophyll content, 80.7% of that from C8 soybean plants inoculated with the wild-type strain (*P* < 0.0001), which was not significantly different from the uninoculated control (Table 1 and S7 Fig). However, the same Δ*znuA* mutant was still fully effective in supporting the growth of W05 (Table 1 and S7 Fig). The mutant for *mdtA*, which is found together with *mdtB/C* in an operon that encodes a putative multi-drug efflux system, was ineffective on both W05 and C08 as indicated by the significantly reduced chlorophyll content of these host leaves compared to those from plants inoculated with the wild-type strain (Table 1 and S7 Fig). Notably, the *mdtA* mutant induced many root bumps on C08 but not on W05 (S7 Fig) and the *mdt* operon is present in CCBAU45436 but not in CCBAU25509 (i.e. it is in subset IV). Both *znu* and *mdt* operons are located on the chromosome.

**Table 1:** Responses of soybean plants to inoculation with *Sinorhizobium fredii* CCBAU45436 and its derivatives.

Inoculant	Number of nodules (/plant)		Nodule fresh weight (mg/plant)		Nodule fresh weight (mg/nodule)		Leaf chlorophyll content (SPAD)	
	W05	C08	W05	C08	W05	C08	W05	C08
Uninoculated control	-	-	-	-	-	-	30 ± 2***	33 ± 3**
wild type	9 ± 4	25 ± 8	50 ± 8	252 ± 51	6 ± 1	11 ± 1	41 ± 2	36 ± 2
*mdtA*::pVO	12 ± 2	13 ± 5**	91 ± 14***	146 ± 56***	8 ± 0	12 ± 1	27 ± 2***	28 ± 3***
Δ*znuA*	4 ± 1**	9 ± 4***	61 ± 12*	146 ± 43***	16 ± 3*	19 ± 2**	40 ± 2	29 ± 3***

Note: multiple independent experiments were carried out and mean ± SD scored from nine plants in the same experiment is shown. Significant difference compared to values of wild type strain are indicated by stars (*, *P* < 0.05, **, *P* < 0.01; ***, *P* < 0.001; Student’s t-test).

## Discussion

### Optimization of symbiotic efficiency requires coordinated regulation of genes from multiple replicons

The transferable symbiosis island or symbiosis plasmid is the major reason for an ever increasing collection of rhizobial germplasm associated with diverse legumes [8–11,37]. The increased contribution of genes on symbiosis plasmids and dramatically reduced contribution of chromosomal genes to the transcriptomes of nitrogen-fixing bacteroids within nodules were observed for both of the *S*. *fredii* strains in this study (Fig 3 and S2 Fig) and in previous transcriptomic studies of *S*. *meliloti* 1021 and *S*. *fredii* NGR234 [24,38,39]. Notably, genes on the symbiosis plasmids of CCBAU45436 and CCBAU25509 that were highly expressed (*Level_4*) in nodules included genes belonging to pangenome subsets I-IV (Fig 3 and S2 Fig). These findings support a model that the symbiosis plasmid harbors genes of different conservation levels that contribute to symbiotic adaptation.

However, a higher level of between-replicon connectivity than within-replicon connectivity was observed for symbiosis plasmids in the co-expression networks (Fig 7B and S4 Fig). Key genes involved in nitrogen fixation (*nif/fix*) have a considerable degree of both within- and between-replicon gene connectivity (S5 Fig). Genes involved in inorganic ion transport and metabolism (COG category *P*), and those belonging to the COG category *X* (mobilome: prophages, transposons) were found to be up-regulated within nodules (Fig 5D). Indeed, some transporters provide elements (such as iron, molybdenum, and sulfur) essential for nitrogenase activity [25,40,41]. The high-affinity transporters for phosphate and zinc were required by *S*. *fredii* to effectively fix nitrogen in soybean nodules [25]. Genes encoding these transporters, and many of those directly involved in nitrogen-fixation, such as *nifH/D/K*, belong to COG category *P*. The activation of mobile elements under symbiotic conditions has been widely observed in many transcriptome analyses [24,28,42], and was recently found to have an important role in the adaptive evolution of rhizobial symbiotic compatibility [17].

The conserved *znu* and accessory *mdt* of CCBAU45436 contributed to symbiotic adaptation to *G*. *max* C08, but to a lesser extent to the symbiosis with *G*. *soja* W05 (Table 1 and S7 Fig). The zinc transporter encoded by *znu* can import zinc under low-zinc conditions [43,44]. This indicates possibly different nodule environments of W05 and C08 with respect to the zinc ion concentration. Although the *mdtA* mutant did not induce pseudonodules (root bumps) on W05 (S7 Fig), *mdt* contributed to the symbiotic efficiency of CCBAU45436 on W05 (Table 1). A reasonable explanation might be that genes other than *mdt* have been recruited by CCBAU25509 to optimize its symbiosis with W05. This view is supported by our recent finding that strain-specific accessory genes can be recruited by different *Sinorhizobium* strains in optimization of symbiosis with the same legume host [27]. Since both *znu* and *mdt* are located on the chromosome, this suggests that chromosomal core and accessory genes can be recruited by *S*. *fredii* to optimize the symbiotic functions in a host-dependent manner. These results increase our understanding of the integration of key symbiosis genes with the diverse genomic backgrounds of rhizobia as characterized by their large phylogenetic diversity [31,32].

### Chromids have a high level of cross-regulation with chromosomes and contribute to environmental adaptations and diversification of *Sinorhizobium*

Co-expression analysis of the two *S*. *fredii* strains under different conditions unveiled a higher level of gene connectivity between chromids and chromosomes than that between symbiosis plasmids and chromosomes (Fig 7B and S4 Fig). This is in line with the computational prediction of the regulatory network in *S*. *meliloti*, i.e. the preference for cross-regulation between the chromosome and chromid, as opposed to the symbiosis plasmid [45]. A recent study of the *S*. *meliloti* metabolome revealed that removal of the chromid has a larger effect on the metabolome than loss of the symbiosis plasmid [46]. These findings support the hypothesis of the ancient integration of chromid functions with those on the chromosome [13]. Indeed, some essential genes can be found on the chromid, but not on the symbiosis plasmid, of *Sinorhizobium* strains [16,47,48]. Moreover, in contrast to genes on symbiosis plasmids, chromid core genes are more likely to have a congruent phylogeny with that of the species tree of *Sinorhizobium* [21].

It was reported that chromids contribute to the intraspecies differentiation of *S*. *meliloti* strains [22]. This is in line with the enrichment of strain-specific genes (subset IV) on chromids of the two *S*. *fredii* strains. Here we reveal that the chromid gene pool also makes a significant contribution to inter-species differentiation in *Sinorhizobium*, as approximately 38.7% of the subset II are located on the chromids of *S*. *fredii*. When the transcriptional profiles of single-copy genes were compared between CCBAU45436 and CCBAU25509, DEGs were significantly enriched on chromids under all test conditions (Fig 6B). It has been demonstrated in *Escherichia coli* that strain-dependent DEGs were more polymorphic or divergent than other genes, indicating the role of differential gene regulation in bacterial diversification [49,50]. These findings indicate that the expression pattern of genes on chromids may evolve relatively rapidly, which echoes a report that genes evolve faster on chromids than on chromosomes [51].

Those genes up-regulated at stationary phase were enriched on chromids of the two *S*. *fredii* strains, and were over represented with genes of unknown function and those involved in modifying extracellular structures, indicating a role of chromids in stress adaptation. Notably, the average level of gene connectivity for chromid genes was generally lower than that for those from chromosome and symbiosis plasmids under test conditions (Fig 7A and S4 Fig). This may be due to a critical role of chromids in intra- and inter-species diversification and in adaptation to more diverse niches [15,16] that were not effectively covered in this study. In line with this view, the chromid of *S*. *meliloti* was enriched with genes that were up-regulated under osmotic stress conditions [52]. Moreover, genetic and metabolic modelling studies show that the chromosome alone is sufficient for the growth of *S*. *meliloti* in sterile soil, while the chromid may confer more specialized functions in the rhizosphere [15,16]. Likewise, among six extrachromosomal replicons including the symbiosis plasmid pRL10 of *R*. *leguminosarum* Rlv3841, many genes of pRL8 are specifically up-regulated in the rhizosphere of pea, but not in that of alfalfa and sugar beet [14], indicating a contribution by pRL8 to host-specific fitness. Therefore, in addition to the well-known symbiosis plasmid essential for symbiotic adaptation, extra-chromosomal replicons including chromids may offer rhizobia novel adaptations that are needed in soils and rhizospheres characterized by highly fluctuating levels of nutrients and stress factors.

### The more conserved a gene is, the greater its level of coordinated adaptive regulation

The transcriptional profiles of pangenome subsets I-III exhibited a strong condition-dependent clustering pattern (Fig 4A–4C) rather than a strain-dependent one as observed for the subset IV (Fig 4D). These results are consistent with the recent comparative transcriptomic analyses of *E*. *coli* strains under free-living conditions, which revealed that the gene expression distances of core genes between strains were mainly dependent on the culture conditions rather than phylogenetic relatedness [50], though a later independent study also identified a large number of strain-dependent transcripts in addition to condition-dependent ones [49]. Distinct characteristics of test conditions among different studies may exert variable strength of influence on clustering patterns.

Earlier transcriptomic studies of *E*. *coli* strains under free-living conditions revealed a positive correlation between ortholog frequency (% *E*. *coli* genomes exhibiting gene) and expression level [50]. In our study, the average expression level of a gene under each test condition (free-living or symbiotic) is positively related to its conservation level in four hierarchical subsets of the *S*. *fredii* pangenome (Fig 4E) from strain-specific to genus core. The most recently acquired genes, such as those of subset IV, showed the lowest variation in expression levels between different conditions, whereas the more conserved subsets III, II and I exhibited increasing expression plasticity (Fig 4F). Moreover, the more conserved a gene is, the higher its level of gene connectivity in the co-expression network (Fig 7A and S4 Fig). These findings highlight that transcriptional regulation contributes to the development of the more conserved pangenome subsets, and the newer pangenome members are less intensively integrated with the core regulation network involved in environmental adaptations. It has been hypothesized that the prokaryotic pangenome mainly results from adaptive, not neutral, evolution [4], and this appears to be true at least for the subsets I-III of the *S*. *fredii* pangenome. For those newly acquired genes with few interaction partners in the pangenome, earlier bioinformatics analysis suggests that they may take many million years to be integrated into regulatory interaction networks [53].

### Conclusion

Prokaryotic core and accessory genome components are analogous to the operating system and applications (apps) of smartphone [54]. This work provides further evidence of the organization, regulation and integration of apps with the operating system in the prokaryotic multipartite genome of *S*. *fredii*. We demonstrated that the average level of gene expression, the variation of gene expression between environments, and the gene connectivity degree within co-expression networks are positively related to the conservation level of a gene. There are replicon biases in genes of different conservation levels, in genes up-regulated under specific conditions, and in the connectivity of genes within co-expression networks. Moreover, chromosomal loci *znu* and *mdt* operons were identified as novel players in host-specific adaptations, which are generally thought to be the domain of the symbiosis plasmid. These findings shed new light on our understanding of the coordinated regulation of core and accessory genes of rhizobia, facultative microsymbionts of legumes. Similar strategy can be used to study other prokaryotes, which are subject to diverse stimuli in the ever-changing circumstances.

## Materials and Methods

### Growth conditions for bacterial strains and plants

*S*. *fredii* strains were cultured at 28°C in tryptone-yeast extract (TY) medium [55], and *E*. *coli* strains at 37°C in Luria-Bertani (LB) medium. When required, the media were supplemented with the appropriate antibiotics at final concentrations of 30 μg/ml for nalidixic acid, 10 μg/ml for trimethoprim, 10 μg/ml for tetracycline, 50 μg/ml for kanamycin, and 30 μg/ml for gentamicin.

Plant growth and inoculation was performed according to the method previously described [25]. Seeds of *G*. *max* C08 were surface-sterilized by successive treatments with 95% ethanol for 30 sec and 3% (w/v) NaClO for 5 min, and were then washed 6 times by autoclaved deionized water. For seeds of *G*. *soja* W05, a pre-treating step in concentrated sulfuric acid for 2 min was needed before the surface-sterilization. The surface-sterilized seeds were germinated on 0.6% agar plates in the dark at 28°C for 36–48 hours. Then, germinated seeds were planted in vermiculite wetted with low-N nutrient solution in Leonard jars [56] and were inoculated with 1 ml of physiological saline suspension (OD_600_ = 0.2) of rhizobia per plant. Plants were grown at 24°C with 12-h day and night cycles for 30 days. Nodules for bacteroid isolation or RNA extraction were harvested, immediately frozen in liquid nitrogen, and then stored at -80°C until use.

### Genome sequencing, assembly and annotation

Illumina paired-end sequences have been previously obtained for the genomes of *S*. *fredii* strains CCBAU45436 and CCBAU25509 [26]. In this study, PacBio and Ion Torrent sequencing technologies were used to get sequences of larger genomic libraries of these two strains (S1 Table). Error correction and a hybrid model were used to perform genome assembly by Celera Assembler V8.3 [57]. Sanger sequencing of PCR products was then used to close sequence gaps. Gene prediction and functional annotation were performed by RAST [58] and Blast2GO [59].

### Comparative genomics analyses

In this study, twelve *Sinorhizobium* genomes, spanning five *S*. *fredii* strains (CCBAU45436, CCBAU25509, HH103, NGR234 and USDA257), six *S*. *meliloti* strains (Rm1021, AK83, BL225C, GR4, Rm41 and SM11) and one *S*. *medicae* strain (WSM419), were used for comparative genomics analyses. Protein sequences encoded by these genomes were collected and clustered by CD-HIT [60] to generate a (0, 1)-matrix describing the distribution of all gene orthologs (>70% identity over at least 80% of the length of the smallest protein) in the pangenome of twelve *Sinorhizobium* strains. Based on this matrix, the core and accessory genomes of *S*. *fredii* CCBAU45436 and CCBAU25509 were defined at three different levels: between CCBAU45436 and CCBAU25509, among *S*. *fredii* strains, and among *Sinorhizobium* strains (S1 Fig). Using this information, the genomes of *S*. *fredii* CCBAU45436 and CCBAU25509 were divided into four hierarchical core/accessory subsets (Fig 2A).

### RNA extraction, library construction and sequencing

Free-living bacterial cultures in TY medium at mid-log phase (OD_600_ = 0.6) and stationary phase (OD_600_ = 4.5) were harvested by centrifugation at 4°C and 12,000 rpm for 10 min. Bacterial RNA extraction was performed using RNApure Bacteria Kit (CWBIO) according to the manufacturer’s recommendation. Bacteroids were isolated from nodules using a method described earlier [24] and ground in liquid nitrogen before RNA extraction. Total RNA from nodules (a mixture of plant and bacterial RNA) induced by CCBAU45436 was also extracted using the TAKARA RNAiso plus reagent.

Strand-specific RNA sequencing was carried out by BGI-Shenzhen with Next Generation Sequencing (NGS). In brief, the integrity and quality of all RNA samples were checked with Agilent Bioanalyzer 2100 (Agilent Technologies). Genomic DNA contamination was removed by DNase I digestion (30 min at 37°C). Total RNA was then treated with the Ribo-Zero rRNA removal kit to remove the ribosomal RNA. The ribosomal RNA-depleted samples were then used to construct whole transcriptome libraries following the manufacturer's instructions (Illumina) and the resultant products were sequenced on an Illumina Hiseq 2000 platform (Illumina). Two independent cultures and two sets of nodules were used to prepare RNA samples.

### RNA-seq reads mapping, DEG calling and gene expression plasticity analysis

Clean reads in fastq files were mapped to the reference genomes of *S*. *fredii* CCBAU45436 or CCBAU25509 using Bowtie2 (default parameters) [61]. Summary statistics for the clean reads data and mapping results are shown in S2 Table. The number of mapped reads for each protein-coding gene was extracted from sorted bam files by HTseq-count (-a 0) [62]. DESeq2 was used to identify DEGs (Log_2_R > 1.732 or 1, FDR < 0.001) using raw counts data as input [63]. Strain-specific genes and multi-copy genes shared by *S*. *fredii* CCBAU45436 and CCBAU25509 were omitted when calling DEGs between these two strains. The expression plasticity of a gene was defined as the variance of the Log_2_-transformed RPKM values of this gene across all transcriptomes of each tested strain.

### Gene clustering and co-expression analyses

Dendrograms of samples were built from normalized RPKM data using the dendextend package in R [64], where multi-copy genes were excluded while the values of strain-unique genes were set to zero for the strain that lacked them. This RPKM dataset were first Log_2_-transformed before calculating Euclidean distance between each sample pair and the final hierarchical clustering (hclust, method = average). This RPKM dataset, after the removal of strain-unique genes and the addition of *nifHDK*-1 genes, was also used for a weighted and signed gene co-expression network analysis by using R statistical package WGCNA [65]. The numbers of co-expressed gene pairs with a Pearson’s correlation coefficient (r) above 0.8 were counted to calculate gene degree values. Condition-dependent co-expression groups were divided by k-means clustering of the RPKM dataset of each test strain by using Gene Cluster 3.0 [66]. The two independent RPKM datasets were Log_2_-transformed, filtered (at least two observations > 4 and Max-Min > 1) and centered by gene, respectively, before the final clustering (k = 3).

### Molecular and genetic manipulations

Strains, plasmids and primers used in this study are listed in S7 Table. The schematic diagrams illustrating the construction of the mutants of representative differentially expressed genes are shown in S6 Fig. In brief, the internal DNA fragments of target genes, which could serve as homology arms for exchanging, were amplified by PCR amplification and each cloned into pVO155, a plasmid used for gene inactivation via site-specific insertion [67]. The resulting pVO155 derivatives were then conjugated into *S*. *fredii* CCBAU45436 and insertion mutants were screened on the TY-agar plates supplied with 30 μg/ml nalidixic acid and 50 μg/ml kanamycin and verified by colony PCR and Sanger sequencing.

### Statistical analyses

All enrichment analyses used in this study were performed by using the Pearson’s chi-square test, and the Benjamini-Hochberg FDR controlling procedure was used for *P*-value correction in multiple comparisons. Correlations were determined with the cor.test R command using the nonparametric Kendall’s s statistic. Two-tailed Student’s t-test was used to compare the symbiotic phenotypes between the wild type and mutant strains.

## Supporting information

S1 Table Sequencing information of three platforms. (XLSX)

S2 Table General features of *S*. *fredii* CCBAU25509 and CCBAU45436 genomes. (XLSX)

S3 Table Biased distributions of core and accessory genes regarding replicons. (XLSX)

S4 Table An overview of RNA-seq reads mapping. (XLSX)

S5 Table Enrichment analysis of condition-dependent up-regulated and down-regulated genes in core/accessory subsets of *S*. *fredii* CCBAU45436 and CCBAU25509. (XLSX)

S6 Table Biased distribution of differentially expressed genes between CCBAU45436 and CCBAU25509 under free-living and symbiotic conditions regarding replicons and hierarchical core/accessory subsets. (XLSX)

S7 Table Strains, plasmids and primers used in this study. (XLSX)

S8 Table Responses of soybean plants to inoculation with *S*. *fredii* CCBAU45436 and its ten mutants. (XLSX)

S1 Fig The pangenome at the intra-species and intra-genus levels. **(A)** Venn diagram showing the numbers of unique orthologous genes in CCBAU45436 and CCBAU25509, respectively, and the number of core orthologous genes found in both strains. **(B)** Flower plot showing the numbers of strain-specific unique orthologous genes, and the number of *S*. *fredii* core orthologous genes. **(C)** Flower plot showing the numbers of strain-specific unique orthologous genes, and the number of *Sinorhizobium* core orthologous genes. The RefSeq genome assembly accession: GCF_000283895.1 (HH103), GCF_000018545.1 (NGR234), GCF_000265205.3 (USDA257), GCF_000006965.1 (Rm1021), GCF_000147795.2 (AK83), GCF_000147775.2 (BL225C), GCF_000320385.2 (GR4), GCF_000304415.1 (Rm41), GCF_000218265.1 (SM11), GCF_000017145.1 (WSM419).
(PDF)

S2 Fig Replicon-dependent transcriptional profiles of genes within different hierarchical core/accessory subsets under free-living and symbiotic conditions. Bar intensities represent the proportion of genes transcribed at four expression levels (arbitrary cut-offs at the first, second and third quartiles of expression profiles based on RPKM values of all genes) for each of the hierarchical core/accessory subsets (I-IV) under free-living (mid-log phase and stationary phase) and symbiotic conditions (bacteroids isolated from *G*. *soja* W05 nodules and *G*. *max* C08). Q1, Q2 and Q3 represent the first, second and third quartiles, respectively. Results are shown separately for each of the five replicons (chromosome, chromid, symbiosis plasmid, pSF45436d, pSF45436e) in the genome of CCBAU45436. Log-linear analyses indicate significant differences among the replicons in the distribution of genes of different transcriptional levels for each of the hierarchical core/accessory subsets under each condition (all *P* < 0.001).
(PDF)

S3 Fig Condition-dependent enrichment of differentially expressed genes in hierarchical core/accessory subsets. All of the differentially expressed genes based on pairwise comparisons are defined as up-regulated genes of condition.column/condition.row (or equivalent to down-regulated genes of condition.row/condition.column; log_2_R > 1.732, FDR < 0.001). Significant enrichment/depletion are indicated by orange/green color (Pearson’s chi-square test, all *P* < 0.05).
(PDF)

S4 Fig Connectivity analyses of gene co-expression networks in the multipartite genome of *S*. *fredii* CCBAU45436. **(A)** Gene connectivity degrees of the hierarchical core/accessory subsets for each replicon. Error bars represent standard error of mean. **(B)** Within- and between-replicon gene connectivity. The total number of gene connectivity identified for each replicon is shown in brackets. The relative abundances of within- and between-replicon gene connectivity are indicated by different sections of the perimeter colored according to the connected replicons (orange, the gene connectivity to the chromosome cSF45436; blue, the chromid pSF45436b; light green, the symbiosis plasmid pSF45436a; dark green, pSF45436d; purple, pSF45436e). Between-replicon gene connectivity is depicted in grey. Multi-copy genes except one out of two *nifHDK* copies were not included in these analyses.
(PDF)

S5 Fig Within- and between-replicon gene connectivity related to genes on the symbiosis plasmid in the co-expression networks constructed by using WGCNA. Multi-copy genes except one out of two *nifHDK* copies were not included in these analyses.
(PDF)

S6 Fig Schematic diagrams illustrating the construction of mutants derived from *S*. *fredii* CCBAU45436. (PDF)

S7 Fig Symbiotic performance of Δ*znuA* and *mdtA*::pVO mutants derived from *S*. *fredii* CCBAU45436 on *Glycine soja* W05 and *Glycine max* C08. (**A**) Soybean shoot and nodule morphology from the plant inoculated with the corresponding strains. Scale bar = 1 mm (indicates the size of nodules). (**B**) Host plant responses to inoculation of *mdtA*::pVO mutant. The red and yellow arrows in rightmost panel point to normal external morphological nodule and nodule-like bumps. Scale bar = 1 cm.
(PDF)

S1 Dataset Differentially expressed genes of CCBAU45436 bacteroids in nodules of W05 and C08. (XLSX)
