# Supplementary material for: Coordinated regulation of core and accessory genes in the multipartite genome of Sinorhizobium fredii
Source: PLoS Genet. 2018 May 24;14(5):e1007428. doi: 10.1371/journal.pgen.1007428 (PMC5991415; doi:10.1371/journal.pgen.1007428)
Supplement: S1 Fig — (A) Venn diagram showing the numbers of unique orthologous genes in CCBAU45436 and CCBAU25509, respectively, and the number of core orthologous genes found in both strains. (B) Flower plot showing the numbers of strain-specific unique orthologous genes, and the number of S. fredii core orthologous genes. (C) Flower plot showing the numbers of strain-specific unique orthologous genes, and the number of Sinorhizobium core orthologous genes. The RefSeq genome assembly accession: GCF_000283895.1 (HH103), GCF_000018545.1 (NGR234), GCF_000265205.3 (USDA257), GCF_000006965.1 (Rm1021), GCF_000147795.2 (AK83), GCF_000147775.2 (BL225C), GCF_000320385.2 (GR4), GCF_000304415.1 (Rm41), GCF_000218265.1 (SM11), GCF_000017145.1 (WSM419). (PDF) [file pgen.1007428.s009.pdf]

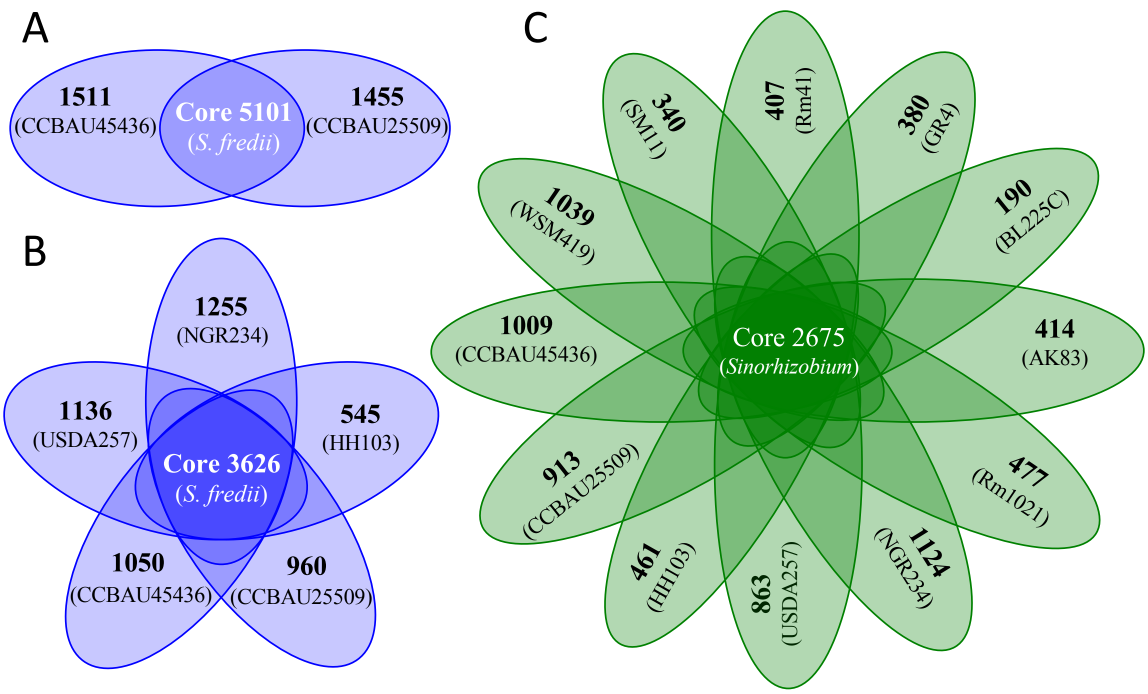

S1 Fig. The pangenome at the intra-species and intra-genus levels. (A) Venn diagram showing the numbers of unique orthologous genes in CCBAU45436 and CCBAU25509, respectively, and the number of core orthologous genes found in both strains. **(B)** Flower plot showing the numbers of strain-specific unique orthologous genes, and the number of *S. fredii* core orthologous genes. **(C)** Flower plot showing the numbers of strain-specific unique orthologous genes, and the number of *Sinorhizobium* core orthologous genes. The RefSeq genome assembly accession: GCF_000283895.1 (HH103), GCF_000018545.1 (NGR234), GCF_000265205.3 (USDA257), GCF_000006965.1 (Rm1021), GCF_000147795.2 (AK83), GCF_000147775.2 (BL225C), GCF_000320385.2 (GR4), GCF_000304415.1 (Rm41), GCF_000218265.1 (SM11), GCF_000017145.1 (WSM419).
