# Supplementary material for: Coordinated regulation of core and accessory genes in the multipartite genome of Sinorhizobium fredii
Source: PLoS Genet. 2018 May 24;14(5):e1007428. doi: 10.1371/journal.pgen.1007428 (PMC5991415; doi:10.1371/journal.pgen.1007428)
Supplement: S2 Fig — Bar intensities represent the proportion of genes transcribed at four expression levels (arbitrary cut-offs at the first, second and third quartiles of expression profiles based on RPKM values of all genes) for each of the hierarchical core/accessory subsets (I-IV) under free-living (mid-log phase and stationary phase) and symbiotic conditions (bacteroids isolated from G. soja W05 nodules and G. max C08). Q1, Q2 and Q3 represent the first, second and third quartiles, respectively. Results are shown separately for each of the five replicons (chromosome, chromid, symbiosis plasmid, pSF45436d, pSF45436e) in the genome of CCBAU45436. Log-linear analyses indicate significant differences among the replicons in the distribution of genes of different transcriptional levels for each of the hierarchical core/accessory subsets under each condition (all P < 0.001). (PDF) [file pgen.1007428.s010.pdf]

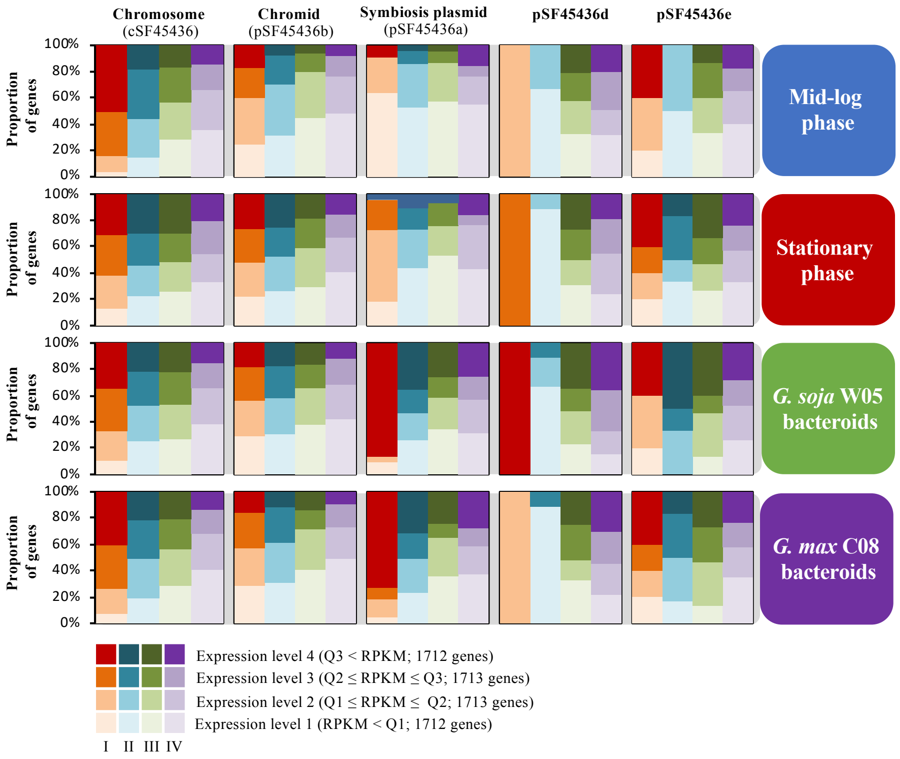

S2 Fig. Replicon-dependent transcriptional profiles of genes within different hierarchical core/accessory subsets under free-living and symbiotic conditions. Bar intensities represent the proportion of genes transcribed at four expression levels (arbitrary cut-offs at the first, second and third quartiles of expression profiles based on RPKM values of all genes) for each of the hierarchical core/accessory subsets (I-IV) under free-living (mid-log phase and stationary phase) and symbiotic conditions (bacteroids isolated from *G. soja* W05 nodules and *G. max* C08). Q1, Q2 and Q3 represent the first, second and third quartiles, respectively. Results are shown separately for each of the five replicons (chromosome, chromid, symbiosis plasmid, pSF45436d, pSF45436e) in the genome of CCBAU45436. Log-linear analyses indicate significant differences among the replicons in the distribution of genes of different transcriptional levels for each of the hierarchical core/accessory subsets under each condition (all $P < 0.001$).
