# Supplementary material for: Coordinated regulation of core and accessory genes in the multipartite genome of Sinorhizobium fredii
Source: PLoS Genet. 2018 May 24;14(5):e1007428. doi: 10.1371/journal.pgen.1007428 (PMC5991415; doi:10.1371/journal.pgen.1007428)
Supplement: S3 Fig — All of the differentially expressed genes based on pairwise comparisons are defined as up-regulated genes of condition.column/condition.row (or equivalent to down-regulated genes of condition.row/condition.column; log2R > 1.732, FDR < 0.001). Significant enrichment/depletion are indicated by orange/green color (Pearson’s chi-square test, all P < 0.05). (PDF) [file pgen.1007428.s011.pdf]

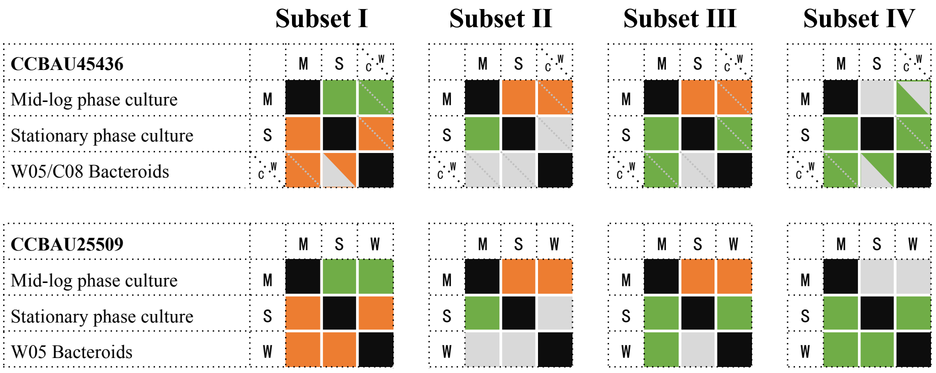

S3 Fig. Condition-dependent enrichment of differentially expressed genes in hierarchical core/accessory subsets. All of the differentially expressed genes based on pairwise comparisons are defined as up-regulated genes of condition.column/condition.row (or equivalent to down-regulated genes of condition.row/condition.column; $\log_2 R > 1.732$, FDR < 0.001). Significant enrichment/depletion are indicated by orange/green color (Pearson's chi-square test, all $P < 0.05$).
