# Supplementary material for: Coordinated regulation of core and accessory genes in the multipartite genome of Sinorhizobium fredii
Source: PLoS Genet. 2018 May 24;14(5):e1007428. doi: 10.1371/journal.pgen.1007428 (PMC5991415; doi:10.1371/journal.pgen.1007428)
Supplement: S4 Fig — (A) Gene connectivity degrees of the hierarchical core/accessory subsets for each replicon. Error bars represent standard error of mean. (B) Within- and between-replicon gene connectivity. The total number of gene connectivity identified for each replicon is shown in brackets. The relative abundances of within- and between-replicon gene connectivity are indicated by different sections of the perimeter colored according to the connected replicons (orange, the gene connectivity to the chromosome cSF45436; blue, the chromid pSF45436b; light green, the symbiosis plasmid pSF45436a; dark green, pSF45436d; purple, pSF45436e). Between-replicon gene connectivity is depicted in grey. Multi-copy genes except one out of two nifHDK copies were not included in these analyses. (PDF) [file pgen.1007428.s012.pdf]

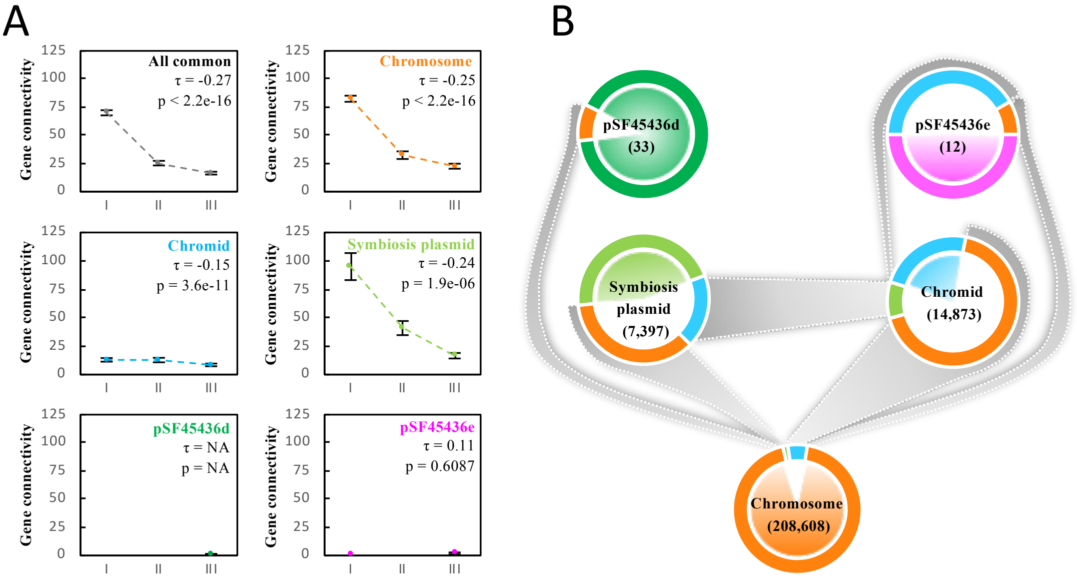

S4 Fig. Connectivity analyses of gene co-expression networks in the multipartite genome of *S. fredii* CCBAU45436. (A) Gene connectivity degrees of the hierarchical core/accessory subsets for each replicon. Error bars represent standard errors of mean. **(B)** Within- and between-replicon gene connectivity. The total number of gene connectivity identified for each replicon is shown in brackets. The relative abundances of within- and between-replicon gene connectivity are indicated by different sections of the perimeter colored according to the connected replicons (orange, the gene connectivity to the chromosome cSF45436; blue, the chromid pSF45436b; light green, the symbiosis plasmid pSF45436a; dark green, pSF45436d; purple, pSF45436e). Between-replicon gene connectivity is depicted in grey. Multi-copy genes except one out of two *nifHDK* copies were not included in these analyses.
