# Supplementary material for: Coordinated regulation of core and accessory genes in the multipartite genome of Sinorhizobium fredii
Source: PLoS Genet. 2018 May 24;14(5):e1007428. doi: 10.1371/journal.pgen.1007428 (PMC5991415; doi:10.1371/journal.pgen.1007428)
Supplement: S5 Fig — Multi-copy genes except one out of two nifHDK copies were not included in these analyses. (PDF) [file pgen.1007428.s013.pdf]

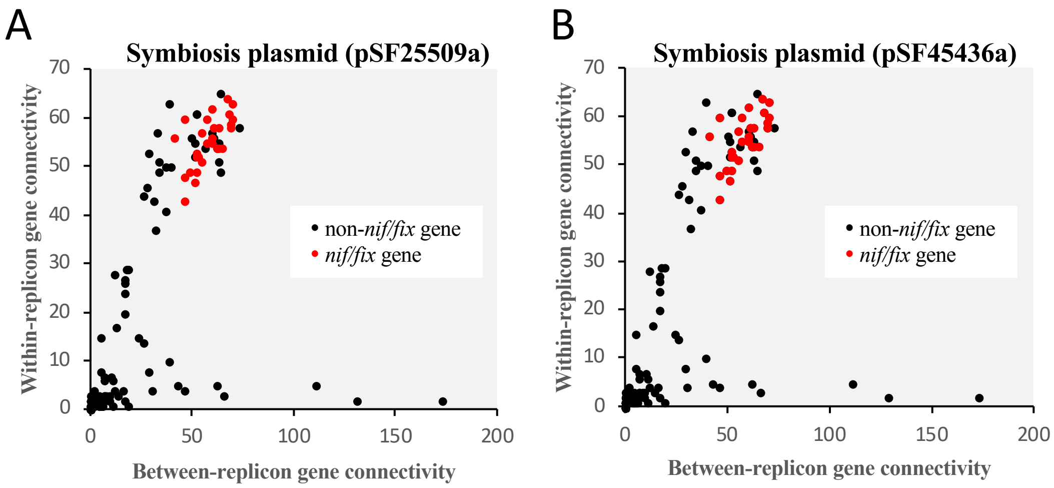

S5 Fig. Within- and between-replicon gene connectivity related to genes on the symbiosis plasmid in the co-expression networks constructed by using WGCNA. Multi-copy genes except one out of two *nifHDK* copies were not included in these analyses.
