# Supplementary material for: Coordinated regulation of core and accessory genes in the multipartite genome of Sinorhizobium fredii
Source: PLoS Genet. 2018 May 24;14(5):e1007428. doi: 10.1371/journal.pgen.1007428 (PMC5991415; doi:10.1371/journal.pgen.1007428)
Supplement: S6 Fig — (PDF) [file pgen.1007428.s014.pdf]

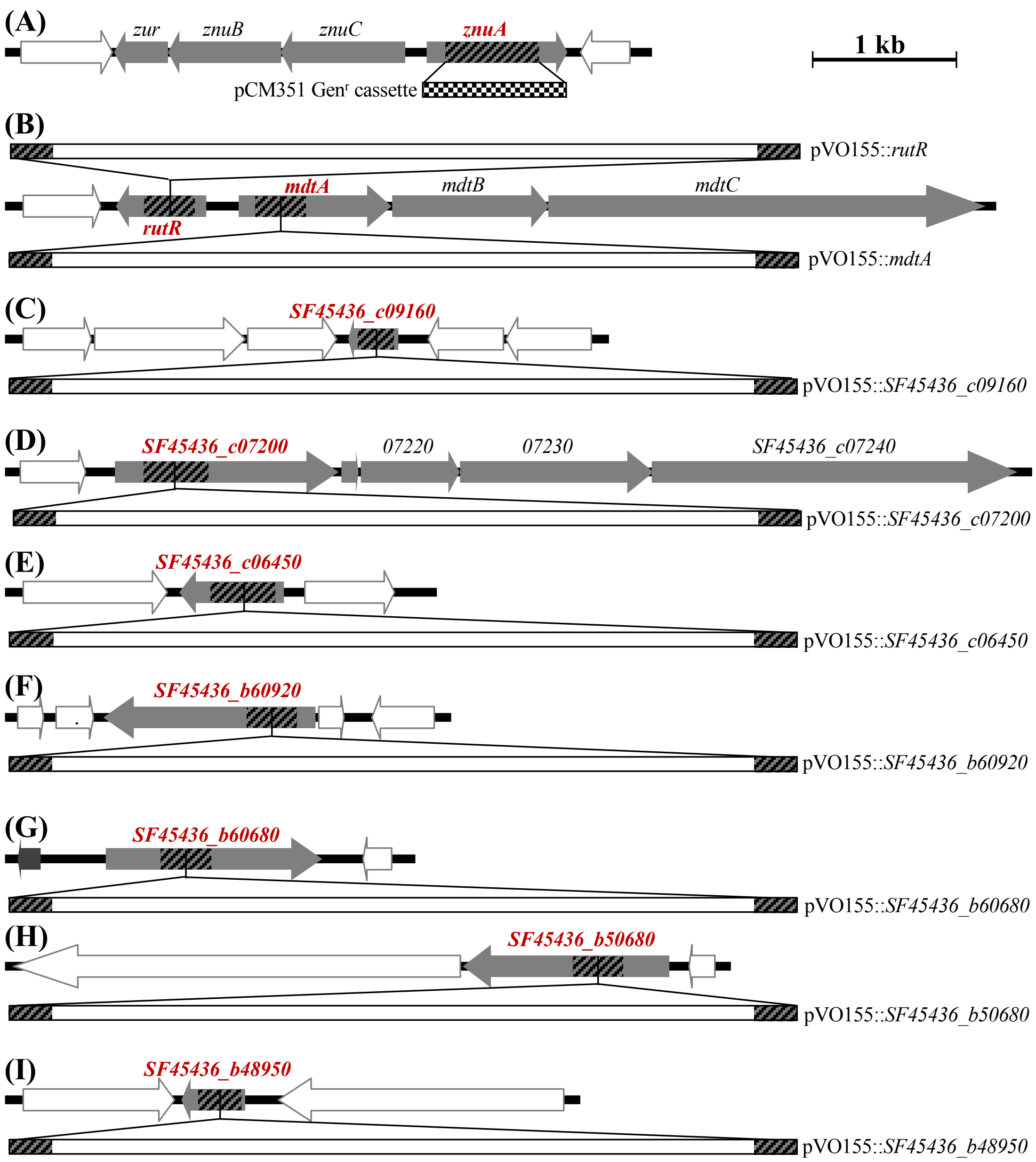

S6 Fig. Schematic diagrams illustrating the construction of mutants derived from *S. fredii* CCBAU45436.
