# Supplementary material for: Coordinated regulation of core and accessory genes in the multipartite genome of Sinorhizobium fredii
Source: PLoS Genet. 2018 May 24;14(5):e1007428. doi: 10.1371/journal.pgen.1007428 (PMC5991415; doi:10.1371/journal.pgen.1007428)
Supplement: S7 Fig — (A) Soybean shoot and nodule morphology from the plant inoculated with the corresponding strains. Scale bar = 1 mm (indicates the size of nodules). (B) Host plant responses to inoculation of mdtA::pVO mutant. The red and yellow arrows in rightmost panel point to normal external morphological nodule and nodule-like bumps. Scale bar = 1 cm. (PDF) [file pgen.1007428.s015.pdf]

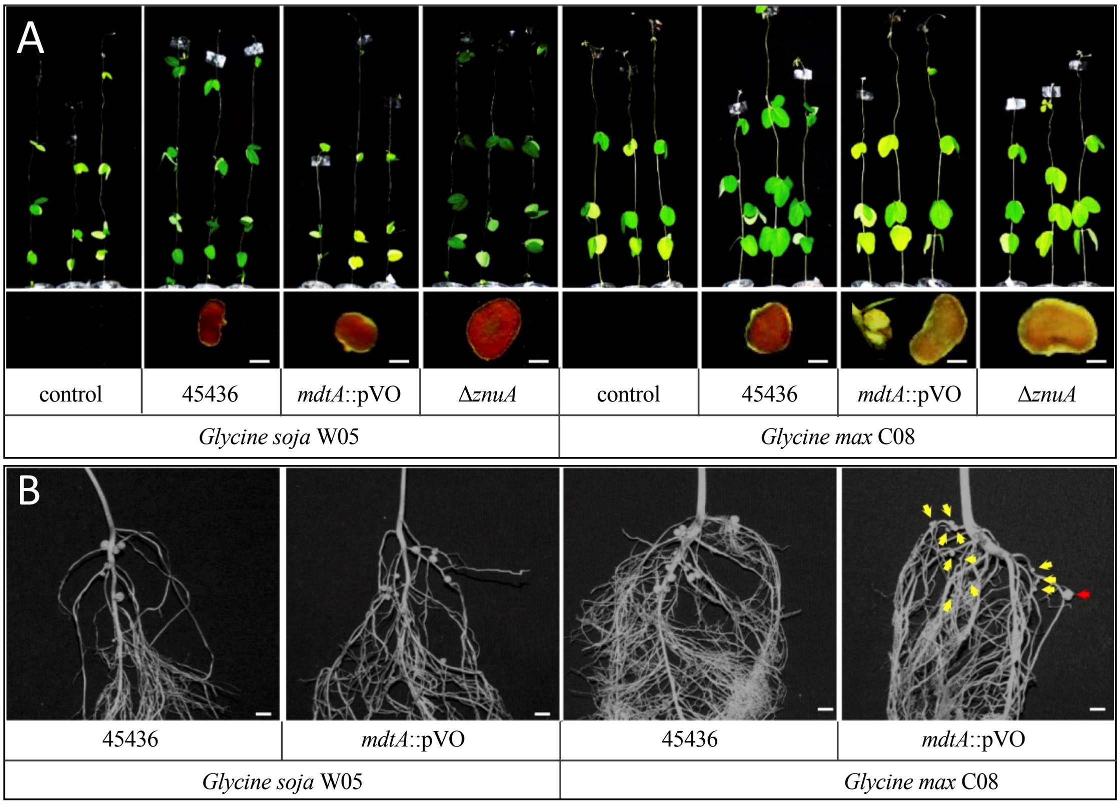

S7 Fig. Symbiotic performance of Δ*znuA* and *mdtA*::pVO mutants derived from *S. fredii* CCBAU45436 on *Glycine soja* W05 and *Glycine max* C08. (**A**) Soybean shoot and nodule morphology from the plant inoculated with the corresponding strains. Scale bar = 1 mm (indicates the size of nodules). (**B**) Host plant responses to inoculation of *mdtA*::pVO mutant. The red and yellow arrows in rightmost panel point to normal external morphological nodule and nodule-like bumps. Scale bar = 1 cm.
